# When medical trainees encountering a performance difficulty: evidence from pupillary responses

**DOI:** 10.1186/s12909-022-03256-3

**Published:** 2022-03-19

**Authors:** Xin Liu, Yerly Paola Sanchez Perdomo, Bin Zheng, Xiaoqin Duan, Zhongshi Zhang, Dezheng Zhang

**Affiliations:** 1 School of Computer and Communication Engineering, University of Science and Technology Beijing, Beijing, 100083 China; 2 Surgical Simulation Research Lab, Department of Surgery, University of Alberta, Edmonton, AB T6G 2E1 Canada; 3 Beijing Key Laboratory of Knowledge Engineering for Materials Science, Beijing, 100083 China; 4 Department of Surgery, Faculty of Medicine and Dentistry, 162 Heritage Medical Research Centre, University of Alberta, 8440 112 St. NW. Edmonton, Alberta, T6G 2E1 Canada; 5 Department of Rehabilitation Medicine, Second Hospital of Jilin University, Changchun, Jilin, 130041 China; 6 Department of Biological Sciences, University of Alberta, Edmonton, AB T6G 2E9 Canada

**Keywords:** Pupillary response, Performance difficulty, Simulation training, Healthcare procedure, Thoracostomy

## Abstract

**Background:**

Medical trainees are required to learn many procedures following instructions to improve their skills. This study aims to investigate the pupillary response of trainees when they encounter moment of performance difficulty (MPD) during skill learning. Detecting the moment of performance difficulty is essential for educators to assist trainees when they need it.

**Methods:**

Eye motions were recorded while trainees practiced the thoracostomy procedure in the simulation model. To make pupillary data comparable among trainees, we proposed the adjusted pupil size (APS) normalizing pupil dilation for each trainee in their entire procedure. APS variables including APS, maxAPS, minAPS, meanAPS, medianAPS, and max interval indices were compared between easy and difficult subtasks; the APSs were compared among the three different performance situations, the moment of normal performance (MNP), MPD, and moment of seeking help (MSH).

**Results:**

The mixed ANOVA revealed that the adjusted pupil size variables, such as the maxAPS, the minAPS, the meanAPS, and the medianAPS, had significant differences between performance situations. Compared to MPD and MNP, pupil size was reduced during MSH. Trainees displayed a smaller accumulative frequency of APS during difficult subtask when compared to easy subtasks.

**Conclusions:**

Results from this project suggest that pupil responses can be a good behavioral indicator. This study is a part of our research aiming to create an artificial intelligent system for medical trainees with automatic detection of their performance difficulty and delivering instructional messages using augmented reality technology.

## Background

Many healthcare procedures involve multiple steps and need to be learned by medical trainees. A trainee is required to learn the procedure step-by-step and correctly follow instructions [1]. Violation of the instructions may lead to unwanted consequences for patient care. To prevent harmful consequences during skill training, these basic procedures are often taught using simulated models [2]. In a routine simulation-based skill training session, clinical instructors are required to be onsite, provide guidance and feedback throughout learning the healthcare procedures. Trainees halt the performance frequently as they need to check the instructions outlined in a textbook or verbally communicate with a clinical instructor standing by. Consequently, the workflow is constantly suspended, and the learning process is interrupted. As the number of trainees and healthcare procedures to be learned are vast, the burden of clinicians engaging in basic teaching is high and often costly.

Our goal was to develop an automatic teaching system that could enhance the learning outcome of the trainees and save the time of clinical instructors in teaching basic healthcare procedures. Such an automatic teaching system could be built with the technology of augmented reality (AR) [3, 4]. In the AR environment, a trainee can see the physical (real) world through a pair of goggles in which the elements are supplemented by computer-generated sensory input such as sound and images. Compared to the traditional way of skills training, AR-aided training offers trainees with instructional messages that can be augmented over surgical sited in the format of text or graphic presentation to save their time in searching for needed instruction [5, 6]. However, these instructional messages are often displayed without mapping to the trainees’ needs. In other words, the augmented message may be presented when a trainee knows how to perform the task. At this moment, it can be a distractor rather than a facilitator [7].

Advances in AR-aided healthcare training systems should find a way to automatically detect the moment of performance difficulty (MPD) so that the artificial intelligent education system can then provide instruction to trainees at the correct moment without disrupting their natural learning process [8]. To achieve this function, the AR training system needs to process behavioral information from learners during skill practice. Here, we need to use an eye-tracker. In surgery, eye-tracking has been gradually applied in training and evaluation [9, 10]. These studies showed that the gaze pattern was different between experts and novices. Trainees can improve their performance and accelerate the learning process via expert-mode visual navigation [11, 12]. The eye-tracker can monitor trainees’ eye behaviors continuously without interfering with their performance in hands. By interpreting eye behaviors, the AR training system may provide usable and reliable instructional information to a trainee at the correct moment without interfering with their learning process [13, 14].

Many signals can be extracted from eye-tracking data; among them, we are interested in pupillary response which can be affected by cognitive activities, perceived workload, and emotional states [15, 16]. Linked to the autonomic nervous system, the quick pupillary response can be observed in 200 ms after mental task change. Kahneman and Beatty suggested that pupil diameter provides a “very effective index of the momentary load on a subject as they perform a mental task” [17]. Preliminary works have also applied several pupillary metrics to measure performance difficulty. For example, peak pupil size increased with surgical difficulty while novices transported rubber objects over dishes with different target sizes and distances [18]; dynamic changes in pupil diameter were performed under conditions of varying cognitive [19].

The above-mentioned information suggests pupillary response can be served as an indicator for the MPD of a trainee when the task difficulty level increased. Research is needed to determine the ability of using pupillary response to detect the MPD of trainees and its value for building a smart AR-aided training system.

We chose thoracotomy in this study for two reasons. First, the procedure of thoracostomy needs to be basic; it should be learned by all medical trainees. As we know, thoracostomy (chest tube insertion) is a daily life-saving procedure that is learned by medical trainees throughout the world. Second, the procedure needs to include multiple steps which will enable us to capture the moment of performance difficulty. A perfect thoracotomy procedure includes eighteen critical steps and medical trainees need to keep these steps in mind and perform each step in order and timely [20]. In this project, we used an eye-tracking-enabled AR platform for recording the eye movement of trainees while they are performing the chest tube insertion.

This study aims to investigate the pupillary response of trainees’ eyes while they encountered the MPD during the learning of a surgical procedure. We hypothesize that a trainee’s pupil dilation will display significant differences during a MPD than a moment of normal performance (MNP). As pupillary responses may differ between an easy and a difficult task, we will adjust pupillary changes by task difficulty. Specifically, we hypothesize:

1. When the participants encounter a MPD, their pupil size will increase as they perceive an increased level of performance difficulty. When the participants seek helps by checking with instructions, their pupil size will decrease as they are releasing mental stresses.
2. The pupillary changes among different performance phases will be influenced by the task difficulty. Exactly, when the participants are performing difficult subtasks, their pupil size will increase more than in performing easy subtasks.

## Methods

### Participants

This controlled laboratory study was conducted at the Surgical Simulation Research Lab of the University of Alberta. The poster of participant recruitment was posted on the designated areas on campus to invite participates. Twelve medical students (50% female, 95% right-handed, age 24 ± 2.7 years) in their first or second year at the University of Alberta were recruited. They were normal (or correct-to-normal) vision and did not have surgical experience.

### Tasks

Participants were required to perform a thoracostomy procedure on the simulation model. The thoracostomy included nine subtasks: (1) identification of landmarks, (2) disinfection, (3) local anesthesia, (4) incision, (5) dissection, (6) insertion, (7) securing, (8) connection to the drainage system, and (9) dressing of the wound. They were required to perform the task as accurately and as fast as possible. According to the hands-on experience from surgeons, subtasks (1)—(4), (8), and (9) were easy, and subtasks (5)—(7) were difficult.

#### Simulation model

**Fig. 1:**
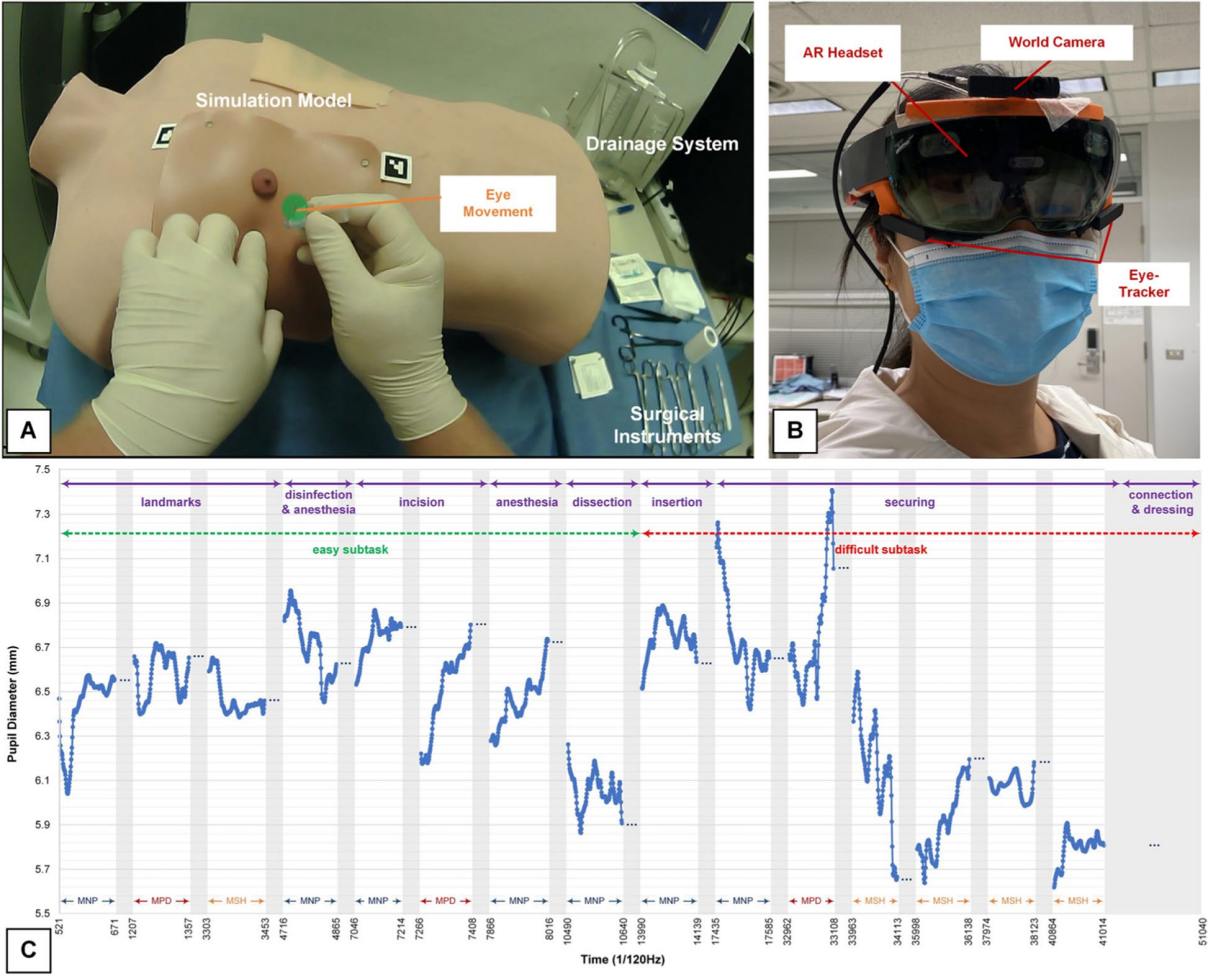
Experimental apparatus. **A** Thoracostomy scenario including simulated human thorax with a skin pad attached with a nipple, surgical instruments, and a drainage system, and the eye movement tracking during the procedure. **B** AR headset with the eye-tracker. **C** Pupil size and phases selection during the thoracostomy procedure

We purchased a standard endurable plastic male torso mannequin (Eddie’s Hang-Up Display Ltd, Canada) with measures of 55 cm length, 48 cm shoulder to shoulder, and 33 cm wide on the chest (Fig. 1A). Part of the right lateral wall of the torso was modified to recreate three average male ribs and their corresponding intercostal spaces. Several 16 cm × 18 cm skin pads were created using customized known materials in the world of simulation (silicones) that replicate human skin sensation and resistance. The skin pads contained three layers: skin, fat, and two-layers muscle.

#### Procedure

Medical trainees have opportunities to observe basic surgical procedures. Before starting the task, subjects were asked to watch a nine-minute demonstration video for the thoracostomy task. The video described detailed steps of chest intubation on a simulated model (plastic male torso, Fig. 1). The procedure was described according to the Advanced Trauma Life Support (ATLS) recommendations. Participants can stop the video at any moment to read the captions, but the video cannot be played back.

Participants were given 20 min to practice hand knots. They could make two consecutive hand knots without assistance which assure that they would complete the suturing step. When ready, participants started to perform the chest intubation on the same simulation model using surgical instruments and a drainage system.

#### Performance

We defined three performances as below to describe the trainees’ operation status. MPD stands for the moment of performance difficulty including mistakes, lapses, or forgetting. The moment of seeking help (MSH) stands for the moment of seeking health information including instructions check and help request. The rest period of time in performing the thoracostomy was called MNP, the moment of normal performance. MNP is selected for each subtask with the following criteria: 1) No presence of MPDs or MSHs; 2) The gaze is on the working site; 3) Events related to instrument handling, such as reaching for the instruments or grabbing the suture with the needle driver, were not included.

The entire surgical procedure may last for 5—10 min. Trainees use most of their time in MNPs, with periodical MPDs and MSHs. On average, each MNP and MPD lasted for 133 and 81 s, respectively. To simplify the data process, we only took no more than 5 s of pupillary data from the middle of each MNP and MPD. Since trainees often dramatically move their heads away from the surgical site during the MSH, we took pupillary data no more than 5 s before the MSH to reduce the variance of pupillary change caused by factors other than surgical performance (Fig. 1C).

### Data recording

All participants wore an AR headset (HoloLens, Microsoft Inc., Redmond, USA) with an embedded eye-tracker (Pupil Core, 120 Hz eye camera, resolution 1920 × 1080-pixel, Pupil Labs Inc., Berlin, Germany) attached to it (Fig. 1B). A specific open-source platform (Pupil Capture 2.3.0) was used to run through the eye-tracker recording to report a list of eye movement data for future analysis. System setup and calibration of the eye-tracker can be found in another research paper [21].

The entire performance video was recorded by a world camera of the eye-tracker, a room camera, and a GoPro action camera (GoPro. Inc., USA) placed inside the mannequin for monitoring the intubation from inside. These videos were used for inspecting the intubation procedures and trainees’ performance.

### Pupillary data analysis

The videos recorded by the world camera of the eye-tracker were analyzed using Pupil Player (Pupil Labs Inc., Berlin, Germany) which can create annotations and trim videos to select the phases for further analysis. Each trial was divided into subtasks and annotated accordingly (identification of landmarks, disinfection, local anesthesia, incision, dissection, insertion, securing, connection to the drainage system, and dressing of the wound). In each subtask, events of interest (MNP, MPD, and MSH) were identified and labeled on the video by the annotation application in Pupil Lab.

#### Pre-processing

The parameter named confidence in the list of eye movement data is an assessment by the pupil detector. A value of 0 indicates no confidence and 1 indicates perfect confidence. In our study, useful raw pupillary data carried a confidence value greater than 0.6 to discard not reliable data. These useful raw pupillary data from the eye-tracker were filtered by a third-order media filter. The range of pupil size observed in all twelve medical trainees is shown in Table 1.

**Table 1:** The range of pupil size was observed in all 12 participants

**Pupil Size**	**subject 1**	**subject 2**	**subject 3**	**subject 4**	**subject 5**	**subject 6**
max (mm)	7.55	7.99	7.99	7.97	6.67	6.64
min (mm)	5.72	5.45	4.70	5.67	4.37	3.76
**Pupil Size**	**subject 7**	**subject 8**	**subject 9**	**subject 10**	**subject 11**	**subject 12**
max (mm)	5.67	7.41	7.77	7.78	7.99	7.99
min (mm)	3.12	5.62	4.80	2.06	5.51	3.95

#### Adjusted pupil size

A subject’s pupil size may dilate during difficult subtasks or encounter MPDs as their stress level increases. The pupil size during a healthcare procedure may be affected by many factors and display enormous individual differences. To make pupillary data comparable, we normalized pupil size for the duration of the experiment expressed as a percentage of the range during the entire procedure. Such an approach can capture similar behavior of the stress response in all trainees. The adjusted pupil size (APS) is:

1 $$\mathrm{APS}=\frac{ps-{ps}_{min}}{{ps}_{max}-{ps}_{min}}\times 100\%$$

where *ps* is the true pupil size, ${ps}_{max}$ and ${ps}_{min}$ are the maximum and minimum pupil sizes during the entire procedure.

In most cases for determining ${ps}_{max}$ and ${ps}_{min}$, the maximum and minimum values were selected from certain periods, such as within MPD, MSH, or MNP; the range of pupillary change (*ps *_*max*_* – ps *_*min*_) was then normalized to [0, 1]. This is a way to make the change of pupil comparable. However, the pupillary range may vary cause by pupil dilation magnitude at different phases. In this study, we determined the *ps *_*max*_ and *ps *_*min*_ from the entire surgical procedure to eliminate potential pupil dilation variation presented at different phases. By keeping the range of pupil change equal, we have increasing confidence to detect different pupillary responses caused by task difficulty and trainees’ performance situations in the study.

Within each phase of MNP, MPD, and MSH, we calculated the maxAPS and the minAPS. As each trainee may have multiple MNP, MPD, and MSH phases, we calculated the meanAPS and the medianAPS for each trainee. We also divided the range of APS (0–100%) equally into 20 intervals with an index from 1 to 20 (e.g. the index 1 represents a 0–5% APS change) to find the index where the APS has the largest difference.

We further compared the cumulative frequency of APS in (35%, 100%], (40%, 100%], (45%, 100%], (50%, 100%], (55%, 100%], and (60%, 100%] and examined at which accumulative frequency of the APS show a significant difference between easy and difficult subtask, and among three different performance situations (MNP, MPD, MSH). Calculating the accumulative frequency is an important step for our succeeding work of applying the deep learning algorithm for automatically detecting the MPD.

### Statistical analysis

On each performance phase ((MNP, MPD, MSH), we recorded data of the maxAPS, the minAPS, the meanAPS, the medianAPS, the max interval index, and the cumulative frequency of APS. The Kolmogorov–Smirnov test showed that the maxAPS, the minAPS, the meanAPS, the medianAPS, and the max interval index were approximately normally distributed (*p* > 0.05); the cumulative frequency of APS did not coincide with a normal distribution (*p* ≤ 0.05).

Our primary goal was to compare the difference in pupillary responses over their different type of performance (MNP, MPD, MSH). We also intended to investigate whether the pupillary difference will be a function of task difficulty. We, therefore, conducted a 2 (task difficulty) × 3 (performance) mixed ANOVA on variables of the maxAPS, the minAPS, the meanAPS, the medianAPS, and the max interval index, with the repeated measures on the second factor. Two separated one-way non-parametric ANOVA (Kruskal–Wallis) were performed on variables of cumulative frequency of APS over task difficulty and three performance situations.

Statistical analysis was performed using SPSS 25.0 (IBM Corp, Chicago, USA). Means and standard errors were reported for significance, with an a priori level of 0.05.

## Results

Thoracotomy videos performed by twelve medical trainees were annotated by an experienced surgeon. A total of 53 MPDs, 124 MSHs, and 81 MNPs were identified from these videos, included in 93 easy subtask’s phases and 165 difficult subtask’s phases. The frequency of MNP, MPD, and MSH in each subtask is shown in Table 2. Pupillary data from these phases were compared.

**Table 2:** Frequency of MNP, MPD, and MSH in each subtask

Subtasks	MNP No. (PCT)	MPD No. (PCT)	MSH No. (PCT)
(1) identification of landmarks	12 (14.81%)	9 (16.98%)	14 (11.29%)
(2)-(3) disinfection & local anesthesia	23 (28.40%)	5 (9.43%)	14 (11.29%)
(4) incision	11 (13.58%)	4 (7.55%)	1 (0.81%)
(5) dissection	11 (13.58%)	8 (15.09%)	19 (15.32%)
(6) insertion	12 (14.81%)	11 (20.75%)	25 (20.16%)
(7) securing	12 (14.81%)	16 (30.19%)	51 (41.13%)
Total (#)	81	53	124
Mean (#/subject)	7	4	10

### Adjusted pupil size

**Fig. 2:**
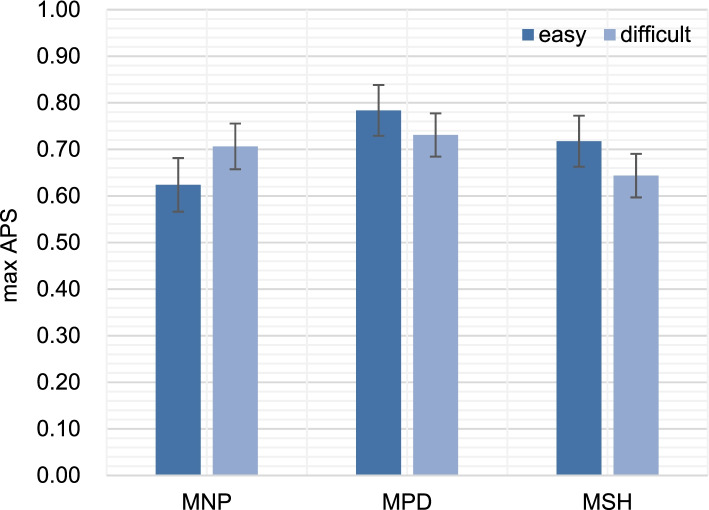
MaxAPS of easy and difficult subtasks in the thoracostomy procedure

The 2 × 3 mixed ANOVA did not reveal any significant difference in APS variables over task difficulty; however, all APS variables showed significant differences in the performance (Table 3). We also found one significant interaction between task difficulty and performance on the measure of maxAPS. As shown in Fig. 2, subjects in MPD displayed a larger maxAPS in MPD than in MNP and MSH; performing difficult tasks did not further enlarge pupil than easy tasks.

**Table 3:** Outputs from 2 × 3 mixed ANOVA

**Parameters**		**Easy**	**Difficult**		***P*-value**
		***Mean* ± *SE***	***Mean* ± *SE***		
maxAPS (%)	MNP	62.38 ± 5.75	70.64 ± 4.91	Difficulty	0.831
	MPD	78.38 ± 5.45	73.09 ± 4.65	Performance	**0.002**
	MSH	71.75 ± 5.49	64.36 ± 4.68	Diffculty * Performance	**0.008**
minAPS (%)	MNP	48.75 ± 5.79	54.55 ± 4.93	Difficulty	0.467
	MPD	50.88 ± 5.13	55.45 ± 4.38	Performance	**0.027**
	MSH	42.75 ± 6.44	47.73 ± 5.49	Diffculty * Performance	0.966
meanAPS (%)	MNP	56.13 ± 6.03	63.18 ± 5.15	Difficulty	0.993
	MPD	68.38 ± 5.16	64.18 ± 4.40	Performance	**0.007**
	MSH	59.13 ± 5.59	56.09 ± 4.77	Diffculty * Performance	0.075
medianAPS (%)	MNP	56.38 ± 6.25	63.36 ± 5.33	Difficulty	0.859
	MPD	69.88 ± 5.48	64.27 ± 4.67	Performance	**0.022**
	MSH	61.13 ± 5.95	56.00 ± 5.08	Diffculty * Performance	0.086
max interval index	MNP	11.83 ± 1.27	13.45 ± 1.08	Difficulty	0.826
	MPD	14.77 ± 1.11	13.24 ± 0.95	Performance	**0.034**
	MSH	12.70 ± 1.22	11.70 ± 1.04	Diffculty * Performance	0.061

### Accumulative frequency of APS change over task difficulty

One-way non-parametric ANOVA on task difficulty revealed significant differences in the cumulative frequency of APS in (45%,100%] (*p* = 0.026), (50%,100%] (*p* = 0.019), (55%,100%] (*p* = 0.044), and (60%,100%] (*p* = 0.032). When performing difficult subtasks, subject displayed smaller accumulative frequency of APS than in performing the easy subtasks (Table 4).

**Table 4:** Cumulative frequency of APS compared between easy and difficult subtasks and among MNP, MPD, and MSH

Parameters	Easy (*n* = 93)		Difficult (*n* = 165)	*P*-value
	*Mean* ± *SE*		*Mean* ± *SE*	
APS in (35%,100%] (%)	94.47 ± 1.89		90.07 ± 1.92	0.412
APS in (40%,100%] (%)	89.63 ± 2.45		84.29 ± 2.40	0.320
APS in (45%,100%] (%)	83.63 ± 3.07		74.65 ± 2.81	0.026
APS in (50%,100%] (%)	73.78 ± 3.95		61.28 ± 3.26	0.019
APS in (55%,100%] (%)	64.60 ± 4.28		50.08 ± 3.45	0.044
APS in (60%,100%] (%)	52.99 ± 4.55		39.72 ± 3.42	0.032
Parameters	MNP (*n* = 81)	MPD (*n* = 53)	MSH (*n* = 124)	*P*-value
	*Mean* ± *SE*	*Mean* ± *SE*	*Mean* ± *SE*	
APS in (35%,100%] (%)	93.60 ± 2.36	95.00 ± 2.39	88.96 ± 2.26	0.007
APS in (40%,100%] (%)	90.19 ± 2.73	91.53 ± 3.36	81.35 ± 2.85	0.014
APS in (45%,100%] (%)	81.99 ± 3.40	85.57 ± 3.91	71.93 ± 3.36	0.019
APS in (50%,100%] (%)	70.16 ± 4.48	74.30 ± 4.82	59.30 ± 3.86	0.067
APS in (55%,100%] (%)	60.07 ± 4.80	62.34 ± 5.75	49.20 ± 3.98	0.105
APS in (60%,100%] (%)	48.51 ± 5.11	52.00 ± 6.01	38.68 ± 3.85	0.092

### Accumulative frequency APS change over performance

One-way non-parametric ANOVA on task difficulty revealed significant differences on the cumulative frequency of APS in (35%,100%] (*p* = 0.007), (40%,100%] (*p* = 0.014), and (45%,100%] (*p* = 0.019) (Table 3). Post hoc analyses were performed to show pairwise comparisons.

For the cumulative frequency of APS in (35%,100%], the differences were presented between MPD and MSH (*p* = 0.008), MNP and MSH (*p* = 0.012), but not between MNP and MPD (*p* = 0.657). When a trainee sought help, their pupils reduced size from the MPD.

For the cumulative frequency of APS in (40%,100%], the differences were presented between MPD and MSH (*p* = 0.014), MNP and MSH (*p* = 0.022), but not between MNP and MPD (*p* = 0.661). When a trainee sought help, their pupils reduced size from the MPD.

For the cumulative frequency of APS in (45%,100%], the differences were presented between MPD and MSH (*p* = 0.017), MNP and MSH (*p* = 0.027), but not between MNP and MPD (*p* = 0.676). When a trainee sought help, their pupils reduced size from the MPD.

## Discussion

This study investigated whether the pupillary response can be used as a behavioral indicator for identifying the MPD of trainees during the thoracotomy procedure. Our first hypothesis was supported by the research results. Compared with normal performance, trainees displayed significantly larger maxAPS, minAPS, meanAPS, medianAPS, and the max interval index when they encountered a MPD. The pupil size increases when the participant encountered a MPD. Once they decided to seek help (MSH), their pupil size reduces. In simple words, the pupillary response provided immediate and spontaneous streams of data for identifying MPDs during healthcare procedures. Instead of reporting the exact pupil size, in this project, we used the APS which pupillary response at any point of time is adjusted by the range of pupil over the entire procedure. In this way, the APS can eliminate the unwanted influence on pupil size while maintaining the power to detect pupillary response as a function of task difficulty and trainee’s performance.

Our second hypothesis was to test whether the trainee’s pupillary response will be regulated by task difficulty. To our surprise in this project, we found that a significant difference was only displayed in the maxAPS between easy and difficult tasks, not in the minAPS, meanAPS, medianAPS, and max interval index [22]. These are not duplicating results from previous studies [15, 23, 24]. A possible explanation is that most of the easy subtasks including identification of landmarks, disinfection, local anesthesia, and incision, are at the beginning of the procedure. The participants of this study were junior medical trainees who were nervous at the beginning of the task performance [25]. When they moved alone to the later stage where subtasks were difficult, they had adjusted themselves by releasing stresses slightly. The easy and difficult subtasks were arbitrated determined by experienced surgeons. To medical trainees, they may feel equally challenging because they are inexperienced in most surgical procedures. These two reasons may diminish the impact of task difficulty on the pupillary response in this study.

There are some limitations to this study. First, eye-tracking data in this study was collected from a controlled simulated environment. Healthcare providers may present different behaviors in the real health environment. Second, the chest tube insertion cannot fully represent the complexity of surgical procedures. Precaution is needed when applying our results to real surgical scenarios. Third, the use of APS for measuring pupillary response has its limitation. The range of pupillary change is determined by the minimal and the maximum values. In a case where a subject’s pupil undergoing a dramatic change due to the factor outside study condition, such as illumination change, our calculation of APS may be affected. The fourth limitation came from our sample size. The number of participants needs to be increased in the future.

Our future goal is to detect the performance difficulty of surgical trainees via a deep learning method. Results from this study suggest that the pupillary response is a promising behavioral marker. We intend to further our research by including more eye-hand coordination data. Once we can detect the MPD, we will build a smart training system to deliver instructional messages to trainees at the right moment to facilitate their skill learning. After this simulation setting, we plan to detect the performance difficulty of surgeons in the operating room. We hope to improve the quality of treatment in real surgery and enhance patient safety with AI technology.

## Conclusion

In conclusion, the pupillary response can help us to identify the moment when medical trainees experienced performance difficulty and intended to seek help during a surgical procedure. Results from this study can inspire our future works by applying the artificial intelligent interpretation of trainees’ performance.

## Data Availability

The datasets during and/or analyzed during the current study available from the corresponding author on reasonable request.

## Abbreviations

APS: Adjusted pupil size
AR: Augmented reality
ATLS: Advanced Trauma Life Support
MNP: Moment of normal performance
MPD: Moment of performance difficulty
MSH: Moment of seeking help
